# Multiple Molecular Pathways in Melanomagenesis: Characterization of Therapeutic Targets

**DOI:** 10.3389/fonc.2015.00183

**Published:** 2015-08-10

**Authors:** Giuseppe Palmieri, MariaNeve Ombra, Maria Colombino, Milena Casula, MariaCristina Sini, Antonella Manca, Panagiotis Paliogiannis, Paolo Antonio Ascierto, Antonio Cossu

**Affiliations:** ^1^Unità di Genetica dei Tumori, Istituto di Chimica Biomolecolare, Consiglio Nazionale delle Ricerche, Sassari, Italy; ^2^Istituto di Scienze dell’Alimentazione, Consiglio Nazionale delle Ricerche, Avellino, Italy; ^3^Dipartimento di Scienze Chirurgiche, Microchirurgiche e Mediche, Università di Sassari, Sassari, Italy; ^4^Istituto Nazionale Tumori “Fondazione Pascale”, Naples, Italy

**Keywords:** molecular melanoma classification, melanoma pathogenesis, signal transduction cascades, targeted-therapy resistance, alternative therapeutic targets

## Abstract

Molecular mechanisms involved in pathogenesis of malignant melanoma have been widely studied and novel therapeutic treatments developed in recent past years. Molecular targets for therapy have mostly been recognized in the RAS–RAF–MEK–ERK and PI3K–AKT signaling pathways; small-molecule inhibitors were drawn to specifically target key kinases. Unfortunately, these targeted drugs may display intrinsic or acquired resistance and various evidences suggest that inhibition of a single effector of the signal transduction cascades involved in melanoma pathogenesis may be ineffective in blocking the tumor growth. In this sense, a wider comprehension of the multiple molecular alterations accounting for either response or resistance to treatments with targeted inhibitors may be helpful in assessing, which is the most effective combination of such therapies. In the present review, we summarize the known molecular mechanisms underlying either intrinsic and acquired drug resistance either alternative roads to melanoma pathogenesis, which may become targets for innovative anticancer approaches.

## Introduction

Melanoma is a heterogeneous disease, with complex pathogenetic mechanisms, as a consequence of specific genetic alterations within several functionally related molecular pathways (1). In fact, studies on genetic and molecular characteristics of melanoma have provided the identification of some specific alterations in pathways controlling cell proliferation, differentiation, and survival. From the practical point of view, increasing evidences indicate that some differences in biological and clinical behaviors within the traditional subgroups of melanomas defined by conventional diagnostic procedures are due to the existence of different “molecular subtypes” of the disease (2). Actually, the criteria commonly used to classify melanomas are based on: (a) relationship between the degree of sun exposition and the site of primary tumor [according to such criteria, melanomas are classified into four groups: melanoma on skin with or without chronic sun-damage (CSD or non-CSD melanoma); melanoma on palms, soles, and nail bed (acral melanoma); and melanoma on mucous membrane (mucosal melanoma)] (1) or (b) evaluation of the tumor growth pattern [according to this criterion, four histological types of melanoma have been described: superficial spreading melanoma (SSM), lentigo maligna melanoma (LMM), nodular melanoma (NM), and acral lentiginous melanoma (ALM)] (3).

Despite the incidence of melanoma has been growing faster than other human cancers during last decades among Caucasian populations (4), most of melanoma cases is diagnosed at early stages of the disease. When patients instead present with an advanced disease (i.e., melanoma is not localized anymore and dissemination of tumor cells to loco-regional or distant sites occurs), very poor survival rates have been reported, due to the lack of effective therapies (3). This, however, happened until few years ago. Very recent advances in molecular oncology have indeed yielded new treatment strategies that target either key effectors of the pathways found to play a major role in the pathogenesis of melanoma – such as those depending on activation of *BRAF*, *NRAS*, or *cKIT* genes – either immune regulatory molecules involved in suppression of the antitumor immune response – such as T-lymphocyte-associated antigen 4 (CTLA4), programed cell death 1 (PD-1), and its ligand (PD-L1) (5).

Although inhibitors of oncogenic *BRAF* generally exert a temporary therapeutic efficacy in patients with metastatic *BRAF*-mutated melanoma, recent evidences seem to indicate that subsets of such cases may present a long-term response to single-agent BRAF inhibition (6). Combination of BRAF and MEK inhibitors has been even proposed as a new targeted-therapy standard of care for BRAF^V600^-positive metastatic melanomas (7).

Monoclonal antibodies directed against the immune ­checkpoints – such as CTLA-4 (ipilimumab), PD-1 (nivolumab and pembrolizumab), or PD-L1 (BMS936559, MPDL3280A, and MEDI4736) – have been demonstrated to achieve durable antitumoral responses with significantly prolonged overall survivals in patients with metastatic melanoma (8). Despite the increased attention is currently paid to cancer immunotherapy (especially, treatments targeting PD-1/PD-L1 molecules) – which may become the standard of care in treating all unresectable stage III and IV melanomas, regardless of the *BRAF* mutational status, we here focused on molecular mechanisms involved in development and progression of the disease. Knowledge of such signaling events may contribute to better define the different subsets of melanoma patients as well as the molecular subtypes participating in response and resistance to targeted therapeutic approaches.

## Mechanisms of Melanomagenesis

### CDKN2A-dependent pathway

**Figure 1:**
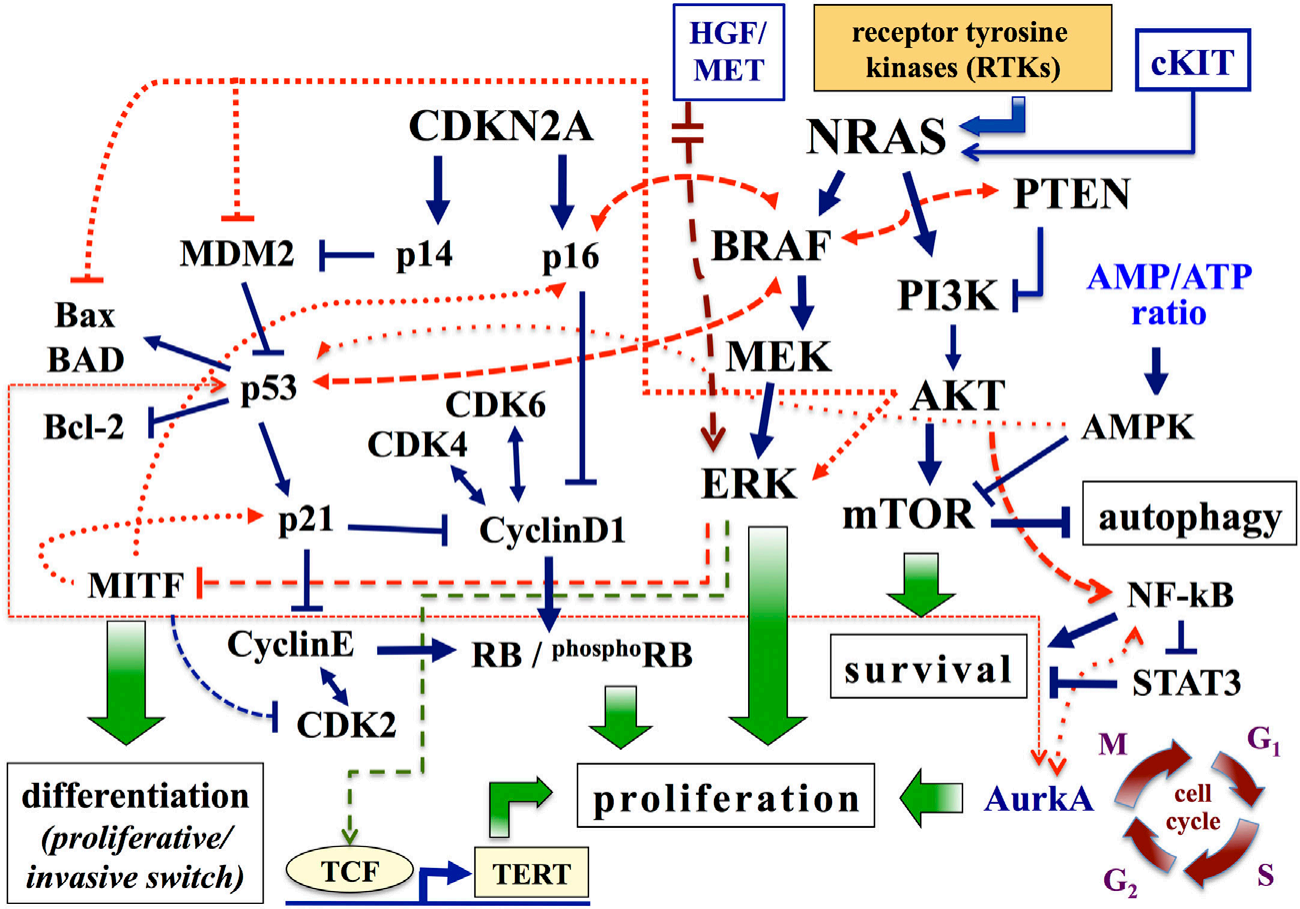
**Major pathways involved in melanoma**. Pathways associated with cell proliferation, survival, and differentiation are schematically presented. Arrows, activating signals; interrupted lines, inhibiting signals. AMPK, AMP-activated protein kinase; Aurk, Aurora kinase; BAD, BCL-2 antagonist of cell death; CDK4, cyclin-dependent kinase 4; CDKN2A, cyclin-dependent kinase inhibitor of kinase 2A; ERK, extracellular-related kinase; HGF, hepatocyte growth factor; MITF, microphthalmia-associated transcription factor; MEK, mitogen-activated protein kinase-extracellular-related kinase; PI3K, phosphatidylinositol 3 kinase; PTEN, phosphatase and tensin homolog; RB, retinoblastoma protein; TERT, telomerase reverse transcriptase.

The cyclin-dependent kinase inhibitor 2A (*CDKN2A*) encodes two proteins: p16^CDKN2A^ and p14^CDKN2A^ (9, 10). *CDKN2A* is a recessive tumor suppressor gene and mutations in this gene are 7–10 times more frequent in patients with a strong family history of melanoma, compared to the vast majority (about 90%) of patients with disease classified as sporadic (11). In physiological conditions, the system p16^CDKN2A^ inhibits protein kinase cyclin-dependent kinase 4 (CDK4)/Cyclin D1 (CCND1), which in turn affects the cell-cycle progression depending on the RB (retinoblastoma susceptibility) protein (12) (Figure 1). Two major alterations reported in melanoma for this pathway are inactivation of p16^CDKN2A^ and amplification of *CCND1*: the first alteration is due to genetic (gene mutations, chromosomal rearrangements) or epigenetic (methylation of promoter regions) mechanisms, while the second one mainly occurs in melanomas negative for mutations in *BRAF* and *NRAS* genes (1, 13). In a small fraction of metastatic melanoma (about 15% of cases), however, *CCND1* amplification and *BRAF* mutations are coexisting and confer resistance to treatment with BRAF inhibitors (14). Similarly, p14^CDKN2A^ interferes with the murine double minute 2 (MDM2) protein, preventing the degradation of the p53 and favoring its control on cell-cycle progression (15) (Figure 1). In melanoma, reducing levels of the p53 protein contributes to boost aggressiveness and refractoriness to therapy; inactivation of p53 can be due to mutations of *p14^CDKN2A^* or to increased expression of MDM2 or, alternatively, to silencing of the *TP53* gene (by epigenetic mechanisms or, to a less extent, sequence mutations) (12, 15).

### MAPK-dependent pathway

The NRAS and BRAF molecules belong to the mitogen-activated protein kinase (MAPK) signal transduction pathway, which mediate the response of cells to mitotic extracellular stimuli and play a central role in regulating cell growth, survival, and cell proliferation. The products of the *RAS* gene family are small proteins bound to the cytoplasmic membrane, with three tissue-specific isoforms: HRAS, KRAS, and NRAS. Among them, *NRAS* mutations are the most detected in melanoma (2, 12, 16, 17). NRAS is able to activate specific cytoplasmic proteins downstream: RAF and phosphatidylinositol 3 kinase (PI3K) (16).

The RAF kinase family consists of three proteins (ARAF, BRAF, and CRAF), whose activation is depending on formation of complexes by these different isoforms (18, 19). All three proteins participate into the transduction of the signal within the MAPK pathway (12, 18). In melanocytes, BRAF induces the activation of MEK kinase, which in turn activates ERK, final effector of MAPK cascade (Figure 1). In melanoma, the *BRAF* gene is mutated in 40–60% of cases; the most prevalent mutation (about 90% of cases) is represented by the replacement of glutamic acid with valine at codon 600 (BRAF^V600E^) (20). The BRAF^V600E^ variant, as the remaining mutations in the BRAF kinase domain, induces continuous stimulation of cell proliferation and tumor growth through activating phosphorylation of ERK. However, the demonstration that *BRAF* is even mutated in common nevi (21) suggests that its oncogenic activation is necessary, but not sufficient, for the development of melanoma. In melanocytes, occurrence of *BRAF* mutations enhances the expression of p16^CDKN2A^ (with normal levels of p14^CDKN2A^), and subsequent induction of cellular senescence and cell-cycle arrest mechanisms. This phenomenon appears as a “protective” reaction, in response to an erroneous mitogenic signal (22). As a confirmation of this, p16^CDKN2A^ expression is reduced or absent in approximately one-third of melanomas with BRAF^V600E^ mutation (22). Similarly, oncogenic activation of *BRAF* is able to promote the malignant transformation of melanocytes deficient in p53 (23). Therefore, BRAF cooperates with members of both pathways controlled by CDKN2A (Figure 1).

### PI3K-dependent pathway

The second pathway depending on RAS for cell growth regulation is constituted by the signal transduction PTEN–PI3K–AKT cascade (16). Under physiological conditions, intracellular levels of PIP2 and PIP3 phosphoinositols are increased by activation of PIK3 and reduced by the activity of the phosphatase PTEN protein (24). High PIP3 levels sequentially activate downstream AKT (mainly, AKT3 in melanoma) and its substrate mTOR, modulating the synthesis of proteins involved in cell growth and survival as well as in apoptosis. In melanoma, *PTEN* gene is deleted in 30–40% of sporadic cases (with loss of the corresponding protein in 5–20% of primary melanomas) and in 30–50% of the cell lines (2, 24). Increased expression of AKT3 is present in 50% of dysplastic nevi, 70% of primary melanomas, and 70% of metastases (24). The activation of AKT: (a) promotes cell proliferation through the induction and stabilization of CCND1; (b) inhibits apoptosis by inactivation of many pro-apoptotic proteins, such as BAD (BCL-2 antagonist of cell death) and MDM2 (which causes the degradation of p53) (1, 12) (Figure 1). In summary, the combined effect of PTEN inactivation and PI3K–AKT stimulation results in an aberrant growth of neoplastic cells, with acquisition of resistance to apoptosis.

### Other proliferation-controlling effectors

Among gene products that operate downstream of the signal transduction BRAF–MEK–ERK pathway, the microphthalmia-associated transcription factor (MITF) seems to play the most relevant role in melanoma (25). In addition to its involvement in skin pigmentation, MITF participates in controlling the proliferation and differentiation of melanocytes (26, 27). MITF activity is complex: a low or absent expression predisposes to apoptosis; intermediate protein levels promote proliferation and cell survival; its overexpression induces cell differentiation and, subsequently, exerts an anti-proliferative effect (25, 27) (Figure 1; for further details, see below). In melanoma, constitutive activation of ERK – mainly, stimulated by oncogenic activation of upstream MAPK components – is associated with a marked degradation of MITF (28). Therefore, the intracellular levels of MITF are dependent on the activation status of the *BRAF* gene. Low intracellular levels of the MITF protein have been reported in invasive melanomas and were associated with a worse prognosis and clinical progression of the disease (29).

Specific sequence variations of cKIT, a tyrosine kinase receptor for stem cell factor, may cause stimulation of the MAPK pathway through constitutive activation of the kinase domain, resulting in induction of cell proliferation (30). In particular, *cKIT* mutations have been reported in acral (10% of cases), mucosal (15–20% of cases), and chronically sun-exposed (5% of cases) melanomas (1, 31). In addition, *cKIT* amplifications or increased gene copies were observed at higher levels in the same series (31).

The pathogenetic scenario previously described cannot be considered inclusive of all molecular alterations that in recent years have been described in melanoma. Among these, it is worth mentioning the alterations of the following effectors: *GNAQ*/*GNA11* genes, which encode signal transmission proteins activating the MAPK pathway, are quite exclusively mutated in uveal melanomas (32); WNT (mainly, the WNT2 isoform), inhibiting apoptosis in melanoma cells and acting as a potential marker of melanocytic malignant transformation (33); iNOS, which regulates the intracellular level of nitric oxide, a free radical involved in the induction of apoptosis, whose increased production can stimulate the development and progression of melanoma (34); NF-kB, which is frequently activated in melanoma, contributing to the disease progression (12); MET, a membrane receptor, activated by binding the hepatocyte growth factor (HGF) ligand, whose increased expression – often due to gene amplification – is involved in enhanced cell invasiveness (35, 36). Moreover, tumor microenvironment (altered distribution and concentration of chemokines, non-activation of cell-mediated immunity, induction of immune-suppressive mechanisms) may play an important role in the formation and maintenance of metastases (37, 38).

## Molecular Subtypes of Melanoma

Considering the distinct molecular pathways as a unique functional network (Figure 1), it becomes clear why changes attributable to the influence of different genes can coexist in melanoma. For example, mutations in *BRAF* can be found associated with alterations in PI3K pathway, but roughly none of them coexist with *NRAS* mutations; since BRAF- and PI3K-driven cascades are activated downstream of NRAS, the presence of activating *NRAS* mutations makes unnecessary the occurrence of BRAF and PI3K activation (39). Similarly, oncogenic mutations in *BRAF* do not fully activate downstream ERK when intracellular mechanisms controlling senescence and/or apoptosis are active (12, 22). Simplifying these complex processes underlying the different phases of disease development and progression, it is possible to select distinct molecular subtypes of melanoma. The characterization of these subtypes becomes extremely important for a more correct therapeutic approach, especially after the introduction of targeted biological therapies into clinical practice.

1. MAPK subtype: mutations in *BRAF* (targetable with specific BRAF-mutant inhibitors or unspecific inhibitors of the downstream activated MEK), which can coexist with other molecular alterations, providing a further stratification of patients:
  - activation of the PI3K–AKT–mTOR system, with increased levels of AKT3 expression and/or loss of PTEN. This subtype may benefit of treatment with inhibitors of PI3K, AKT, and mTOR (40);
  - impairment of the p16^CDKN2A^–CDK4–RB pathway, with inactivation of *p16^CDKN2A^* and/or amplification of *CDK4*. It may be treated with CDK4/6 inhibitors (41);
  - amplification of the *MITF* gene, with or without alterations of the corresponding protein expression levels. It may benefit of treatment with inhibitors of histone deacetylase (HDAC), able to interfere with the expression of MITF protein (42, 43).
2. NRAS subtype: mutations in *NRAS*, playing a crucial role in initiation and promotion of many human cancers, with increased levels of phospho-ERK expression, associated with possible activation of PI3K-AKT pathway (44). This subtype may benefit of treatment with inhibitors of PI3K or MEK (45–47). Poor effects have instead been reported using the specific inhibitors of farnesyl transferase (48, 49).
3. cKIT subtype: mutations in *cKIT*, with or without gene amplification and/or increased levels of protein expression. For this subtype, the cKIT inhibitors are utilized in presence of specific activating mutations (in particular, variants K642E and L576P as well as those in exon 11), which are primarily responsive (50).
4. GNAQ/GNA11 subtype: mutations in *GNAQ* or *GNA11*, with increased levels of expression of phospho-ERK. It may be treated with MEK inhibitors (47).

Molecular analyses have revealed that roughly 50% of melanomas harbor *BRAF* mutations, whereas *NRAS* mutations are observed in 15–20% of them (44, 51). Since *BRAF* and *NRAS* mutations are mutually exclusive in nearly all cases (52, 53), it is widely recognized that about two-thirds of patients present a melanoma with activation of the MAPK pathway, carrying a mutated *BRAF* or *NRAS* gene. The *BRAF* mutations are especially found in younger individuals and in those with melanomas that originate on the skin not chronically exposed to the sun (54). Nearly, all mutations in *BRAF* gene are represented by a substitution of valine at position 600 (V600) (20). Among them, 75% of BRAF variants are represented by the V600E mutation, 19% by the V600K mutation, the remaining 6% by V600D or V600R mutations (55).

The second-generation of RAF inhibitors, specifically targeting the mutated BRAF protein – such as vemurafenib, dabrafenib, and LGX818, potently inhibit MEK phosphorylation and cell growth in *BRAF*-mutated melanomas, being highly effective in inducing rapid tumor regression among melanoma patients (56–58). MEK is a molecular target in MAPK pathway, immediately downstream of BRAF. In experimental cellular models, treatment with inhibitors of MEK is effective on lines mutated in either *BRAF* or *NRAS* (59). Allosteric inhibitors, such as selumetinib and trametinib, and adenosine triphosphate (ATP) non-competitive inhibitors, such as cobimetinib and MEK162, specific for MEK1 and MEK2 kinases are being investigated in *BRAF*- or *NRAS*-mutated cutaneous melanoma as well as in metastatic uveal melanoma (60–62). The *NRAS* mutations are associated with a more aggressive clinical course in melanoma patients (63); tumors carrying such mutations may benefit of the simultaneous inhibition of the MAPK and PI3K pathways (59, 64).

## Resistance to Targeted Therapies in MAPK and NRAS Subtypes

Overall, MAPK and NRAS subtypes represent vast majority of melanoma cases. Tumor responses produced by the main targeted inhibitors in such melanomas are largely partial and tumor resistance typically develops in few months as a consequence of the activation of alternative proliferation-inducing pathways (58, 59). Taking in mind the complexity of the molecular mechanisms involved in pathogenesis of melanoma, it is mostly expected that inhibition of a single component in signaling pathways cannot yield a durable antitumor response and, conversely, that combinations of drugs targeting different key effectors controlling tumor growth may be the solution for a more effective cancer therapy.

**Figure 2:**
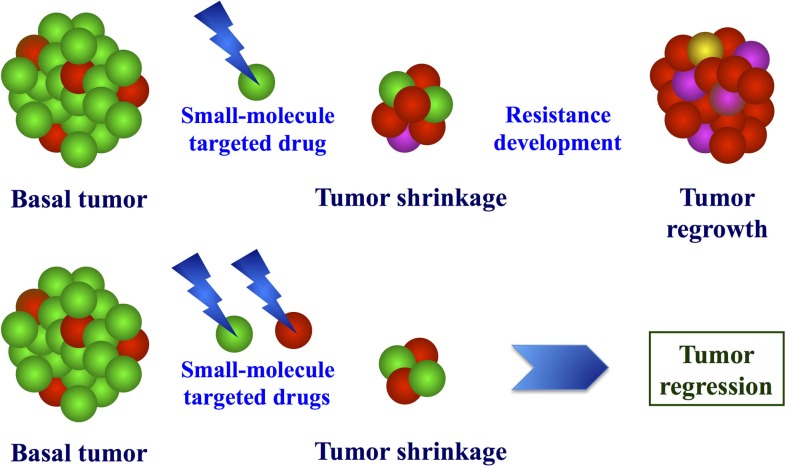
**Model of molecular effects after exposure to targeted drugs**. Due to molecular heterogeneity of melanoma, treatment with single or combined target inhibitors may induce selective pressure of drug-resistant cells (up) or proliferative block of the different cell populations (bottom).

Moreover, several evidences indicate that molecularly heterogeneous cell types may coexist in primary melanomas (65–68). Considering the various pathways involved in melanoma progression as a complex electrical circuit, it is indeed likely that different switches can constitutively activate different molecular processes, which contribute to the different characteristics of malignant cells (survival, independence on apoptotic stimuli, invasiveness, metastatic potential, etc.). When individual pathways are pharmacologically targeted, two scenarios may arise: (a) melanoma cells may selectively activate alternative pathways that allow them to escape the growth block induced by targeted agents; or (b) the selective pressure may induce proliferation of subclones with molecular features different from those presented by vast majority of cell constituting the primary tumor. Recent evidence that the combined use of inhibitors, which simultaneously target multiple pathway effectors, is much more effective than treatment with single or sequentially administered drugs (69–71) strongly supports the latter hypothesis, about the induction of subclonal selection from the heterogeneous tumors undergoing targeted therapies. In Figure 2, the advantage of the initial treatment with multiple target inhibitors is schematized. This is consistent with several indications coming from resistance studies in other malignancies (72–74).

**Figure 3:**
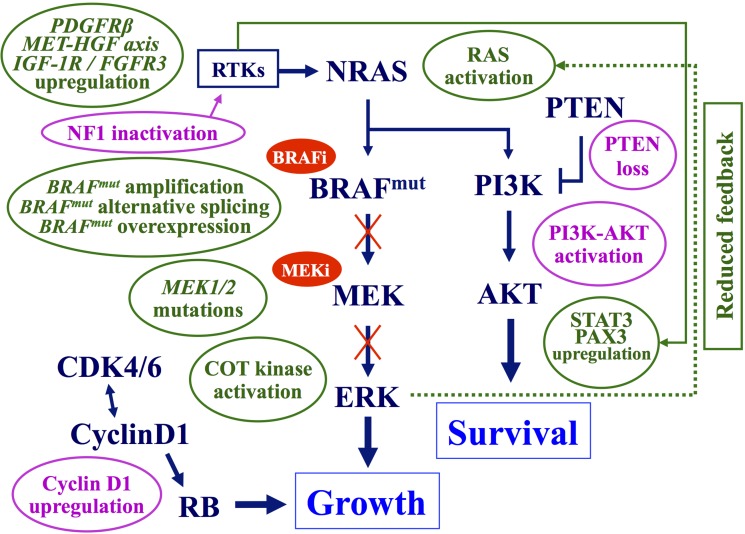
**Main mechanisms of acquired resistance to BRAF–MEK inhibitors**. Preexisting (pink balloons) or acquired (green balloons) mechanisms interfering with the antitumor activity of BRAF and/or MEK inhibitors. Arrows, activating signals; interrupted lines, inhibiting signals. CDK, cyclin-dependent kinase; COT, cancer Osaka thyroid kinase; ERK, extracellular-related kinase; FGFR3, fibroblast growth factor receptor 3; HGF, hepatocyte growth factor; IGF-1R, insulin like growth factor-1 receptor; MEK, mitogen-activated protein kinase-extracellular-related kinase; PAX3, paired box homeotic gene 3; PDGFRβ, platelet-derived growth factor receptor-beta; PI3K, phosphatidylinositol 3 kinase; PTEN, phosphatase and tensin homolog RTKs, receptor tyrosine kinases; STAT3, signal transducer and activator of transcription 3.

To date, several mechanisms of resistance to targeted therapies (mostly, BRAF-mutant inhibitors) have been reported in melanoma (Figure 3).

## Intrinsic Resistance

Lack of antitumor response to commonly used BRAF inhibitors, vemurafenib and dabrafenib, was observed in about one-fifth of treated patients (75). Such an observation is strongly indicative for the existence of an intrinsic resistance to these drugs, which may be due to:

- inactivation of *PTEN* tumor suppressor gene, with subsequent induction of downstream AKT activity (76);
- increased intracellular levels of the cyclin D1 protein (due to gene amplification and/or expression enhancement), which overcome the inhibitory effects exerted by p16^CDKN2A^ kinase and promote stimulation of the RB signaling (14). On this regard, cyclin D1 accumulation in melanoma cells has been recently reported to be partially dependent on inactivation of the FBXO4 gene, which encodes an enzyme regulating the cyclin D1 proteolysis (77);
- silencing of the NF1 gene, which activates RAS and down-regulates the senescence processes controlling cell proliferation (78);
- activation of protein kinase D3 (PRKD3), enhancing the activity of the PI3K–AKT pathway, in response to the inhibition of the oncogenic BRAF (79).

Although apparently unrelated, all these different molecular alterations are able to confer resistance to BRAF or MEK inhibitors in melanoma cells, through their tight interaction with the activity of the RAF–MEK–ERK signaling cascade. Activating mutations in *BRAF* strongly stimulates cell-cycle progression through constitutive stimulation of the downstream MEK–ERK effectors (20). Moreover, activated BRAF usually drives melanocytic proliferation but seems to be insufficient to promote melanoma growth and progression, unless alterations in additional cell-cycle regulating factors – such as p53 impairment, p16^CDKN2A^ loss, increased levels of active AKT – coexist (2, 12).

## Acquired Resistance

Two distinct schemes may be delineated for acquired resistance (Figure 3). The first one is based on changes progressively induced by BRAF inhibition within the same MAPK pathway. Reactivation of MAPK signaling represents the main mechanism of acquired resistance to BRAF inhibitors and it may be achieved through several modifications: MEK-activating mutations, COT/MAP3K8 kinase up-regulation (inducing a MEK–ERK activation, which is thus independent on upstream *BRAF* status), BRAF^V600E^ splicing variants (unresponsive to BRAF inhibitors), *BRAF*-mutated gene amplification (rendering insufficient the BRAF inhibition levels) (56, 75). Both NRAS activation [through acquired mutations and/or functional inductions – such as its reactivation secondary to the reduced negative feedback by active ERK (80)] – and loss of NF1 have also been reported (81). Although NRAS and NF1 act upstream of BRAF in the MAPK pathway, hyperactive NRAS can restore MAPK signaling in the context of BRAF inhibition via paradoxical activation of CRAF (82).

The second scheme includes MAPK pathway-independent mechanisms of acquired resistance, based on rescue of the suppressed ERK activity through changes in alternative pathways controlling cell proliferation (Figure 3). They include activation of the receptor tyrosine kinases (RTKs) – such as the platelet-derived growth factor receptor β (PDGFRβ) (83) or the EPHA2, a member of the RTK subfamily erythropoietin-producing hepatocellular (EPH) receptors (84, 85); activation of the HGF/MET system (86); amplification of the *CCND1* gene – with increased levels of the corresponding protein and subsequent activation of the CDK4/6-RB cascade; lack of the PTEN function (87) or enhancement of the IGF-1R receptor activity (88) – with stimulation of the PI3K–AKT pathway; induction of the complex constituted by the signal transducer and activator of transcription 3 (STAT3) and the paired box homeotic gene 3 (PAX3) effectors – with increased AKT activity (89, 90). In this scheme, BRAF inhibition does not produce a significant antitumor effect, since tumor growth and survival become independent upon RAF–MEK–ERK signaling.

### RAS activation

In about half of melanomas, presence of *BRAF*-mutant monomers determines a constitutive activation of the downstream ERK effector. After BRAF inhibition, ERK signaling is switched off, with a reduced negative feedback on RAS. Vemurafenib and dabrafenib potently inhibit BRAF-mutant monomers and turn down the ERK activity, progressively reducing the negative feedback on RAS-driven signal transduction. The result is a progressive restoration of functional levels of active RAS-GTP, able to induce the generation of RAF dimers, which are resistant to RAF inhibitors. The RAF homodimers (CRAF–CRAF) or heterodimers (BRAF-mutant–CRAF) can re-stimulate the MEK–ERK signaling cascade, with constitutive reactivation of ERK (82, 91). The RAS-driven signal transduction can be also stimulated by activation of the fibroblast growth factor receptor 3 (FGFR3), again conferring resistance to vemurafenib in BRAF^V600E^ melanoma cells (92). Increased intracellular levels of the RAF dimers – as consequence of the activated RAS-driven signal transduction – play also a role into the pathogenesis of squamous cell carcinomas, which represent a peculiar side effect in subsets of patients treated with BRAF inhibitors (93). RAF dimerization is particularly promoted by these agents in cells lacking *BRAF* mutations, leading to induction of keratinocyte proliferation through activation of the MAPK pathway (94–96). Activating mutations in *NRAS* (usually, affecting the codon 61 of the gene) can be also induced by the treatment with BRAF inhibitors (97, 98).

### Changes in BRAF

In a subset of melanomas, amplification of the BRAF-mutant allele – which has been detected at low level in untreated cells – may be induced in response to BRAF or MEK inhibitors, also contributing to resistance to these targeted drugs (99). Moreover, the intracellular accumulation of a splicing variant of the BRAF-mutant mRNA has been described in a subset of BRAF-inhibitor-resistant melanoma cells (82). In fact, such resistant cells express a truncated form of BRAF^V600E^, p61BRAF^V600E^, which lacks the region of RAS-binding domain and results constitutively activated through dimerization of the truncated BRAF-mutant isoforms (82). The final effect is a transactivation of the MEK–ERK signal cascade, with acquisition of resistance to BRAF inhibitors (82). Moreover, the vemurafenib-resistant BRAF-mutant melanomas may acquire dependency on the presence of the targeted drug for their proliferation, such that interruption in administering the BRAF inhibitor may lead to regression of non-lethal drug-resistant tumors (100). On this basis, it has been postulated that a discontinuation in the treatment with these agents may delay the development of lethal drug-resistant cell clones (100).

### ERK activation via alternative kinases

Resistance to BRAF inhibitors may involve an alternative way of stimulation of the ERK signaling. Amplification of the MET receptor and increased levels of its ligand, HGF, play both a role in either intrinsic or acquired resistance to BRAF inhibition (86, 101). In particular, activation of the HGF–MET system strongly stimulates the signal transduction of the downstream PI3K–AKT pathway (101). According to this, administration of a HGF or MET ­inhibitor in combination with a BRAF inhibitor may prevent the resistance induced by using the BRAF inhibitor alone (86).

Other RTKs contribute to anti-BRAF drug resistance. The IGF-1R signaling also activates the MAPK pathway, participating into the different phases of melanocytic transformation and progression (102). Interruption of IGF-1R signaling has been shown to inhibit tumor growth and block metastasis formation in a wide variety of tumor models (88). The activated IGF-1R-dependent signal transduction also targets the PI3K–AKT pathway (102). Coupled inhibition with IGF-1R and MEK inhibitors induce growth arrest in BRAF inhibitor-resistant cells (88).

In a manner independent on the MAPK pathway activation, up-regulation of the PDGFRβ receptor may promote the expression of the transcriptional activation factors STAT3 or PAX3, increasing cell survival and thus reducing the effectiveness of BRAF or MEK inhibition (83, 103). As confirmation of this, inactivation of such genes may restore the block of malignant proliferation in vemurafenib-resistant melanoma cells (89, 90). Down-regulation of STAT3, by BRAF–MEK inhibitors may decrease the activity of anti-apoptotic protein Mcl-1 and reduce melanoma cell survival (104). In contrast, cells presenting up-regulation of STAT3 – as consequence of RTK activation – may acquire independency on activity of the MAPK pathway for their proliferation and survival, with subsequent development of resistance to BRAF and MEK inhibitors (89, 90, 105). Finally, activation of RTK may be also driven by induction of the EPHA2 signaling; in melanoma cells resistant to vemurafenib, EPHA2 overexpression seems to contribute to cell survival and viability as well as to promotion of metastasis formation (84, 85).

Vast majority of data evaluating the role played by RTK-dependent changes in acquired resistance to targeted drugs were produced by *in vitro* studies on melanoma cell lines and awaited to be confirmed *in vivo* on clinical samples.

### Reactivation of MEK-ERK pathway

BRAF or MEK inhibitors may upregulate MAP3K8 gene, giving an overexpression of the corresponding COT kinase, which in turn may stimulate the downstream MEK–ERK signaling cascade and lead to the development of resistance to such drugs (106). Alternatively, acquisition of activating mutations in either *MAP2K1* or *MAP2K2* genes (encoding MEK1 or MEK2 proteins) may directly reactivate the MAPK pathway (107). In particular, mutations in *MAP2K1* gene may constitutively activate the kinase domain of the correspondent MEK1 protein, which therefore becomes either independent on activity of the upstream BRAF kinase either insensitive to MEK inhibitors (75).

Several alterations described for the resistance to inhibitors of mutated BRAF play a similar role in acquisition of resistance to MEK inhibitors. They include BRAF-mutant amplification, STAT3 up-regulation, or MAP2K1/MAP2K2 mutations (75, 105). The activation of the PI3K–AKT pathway also represents one of the main mechanisms of resistance to MEK inhibitors in *BRAF*-mutant melanomas. As confirmation of this, inactivation of such a pathway through up-regulation of the *PTEN* tumor suppressor gene markedly increases the sensitiveness of melanoma cells to MEK inhibitors (108).

### Resistance through phenotype switching

**Figure 4:**
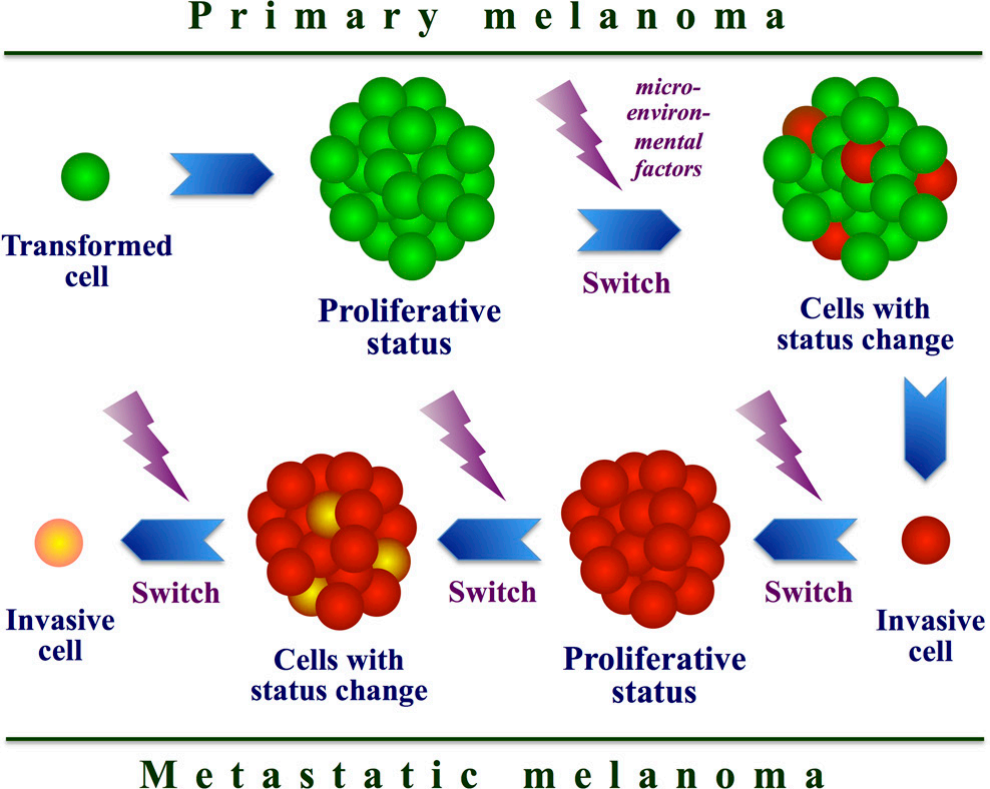
**Model of cell phenotype switching**. Interaction between intracellular and microenvironmental factors may determine a continuous switch from a proliferative to an invasive state in melanoma cells during tumor progression.

Although other MAPK-independent mechanisms have also been identified, resistance still cannot be explained in up to 40% of all patient samples (75, 109), suggesting that additional and perhaps alternative systems, likely involving epigenetic events or stromal factors (101), remain to be identified. Recently, the occurrence of “phenotype switching” has been indicated as an escape mechanism (Figure 4). Essentially by switching from a proliferative to an invasive state, melanoma cells can acquire resistance to targeted therapeutics. Phenotype switching possesses a remarkable similarity to the epithelial-to-mesenchymal-like transition that has been described to occur in cancer stem cells in other tumor types (110) and, interestingly, the characteristics of this phenomenon may provide targets for new therapeutic intervention. The observations on this phenomenon challenged a previous model of melanoma progression that evokes one-way changes in gene expression. Expression profiling of melanoma cell lines identified two transcription signatures, corresponding with proliferative and invasive cellular phenotypes, and *in vivo* melanoma cells may switch between these two states (29). Melanoma cells with proliferative signature are faster growing but less motile than those with invasive signature, being indeed detected most frequently in the peripheral margin of growing tumors. Melanoma cells undergo such a transcriptional signature switching *in vivo*, likely regulated by local microenvironmental conditions (29, 111).

Proliferative profile is based on up-regulation of *MITF* and other melanocytic genes (e.g., *TYR*, *DCT*, *MLANA*) as well as on involvement of additional neural crest-related factors (e.g., SOX10, TFAP1A, and EDNRB) (29, 111). This signature is associated with high rates of proliferation, low motility, and sensitivity to growth inhibition by TGF-beta. The invasive signature down-regulates these same genes and instead up-regulates others ones (e.g., *INHBA*, *COL5A1*, and *SERPINE1*), which are involved in modifying the extracellular environment (112). This second profile is associated with lower rates of proliferation, high motility, and resistance to growth inhibition by TGF-beta. In particular, MITF acts as a master regulator of melanocyte development, function, and survival, by modulating various differentiation and cell-cycle progression genes (28, 113) (see Figure 1). As mentioned above, MITF is amplified in a fraction of human melanomas (28). In addition to transcriptional regulation, MITF is subject to various post-translational modifications, which mainly include MAPK-driven phosphorylations (113). These kinases reside within various important homeostatic signaling pathways and might therefore modulate MITF transcriptional activity in response to specific environmental signals. Genes targeted by MITF can be classified into two groups: differentiation and survival genes. The ability of MITF to regulate cell-cycle progression might also be mediated by modulation of CDK2 or the anti-apoptotic factor BCL2 (114). MITF can act as an anti-proliferative transcription factor able to induce G1 cell-cycle arrest that is dependent on MITF-mediated activation of the p21 cyclin-dependent kinase inhibitor gene (113). MITF cooperates with the retinoblastoma protein Rb1; combination of the MITF-mediated activation of p21 expression and hypo-phosphorylation of Rb1 contributes to cell-cycle exit and activation of a differentiation program. MITF amplification was prevalent in metastatic disease and correlated with decreased overall patient survival. Coexistence of high MITF expression levels and *BRAF* mutations is able to transform human melanocytes; thus, MITF can function as a melanoma oncogene (28, 113). Moreover, a reduction of MITF activity sensitizes melanoma cells to chemotherapeutic agents.

It has been evidenced that melanoma cell lines with high levels of MITF (MITF^high^) were sensitive to either BRAF^V600E^ or MEK inhibition, whereas cells with low MITF levels (MITF^low^) displayed intrinsic resistance against the same drugs (112). Cells with a so-called proliferative signature had a higher proliferation rate and expressed melanocyte differentiation markers, including MITF^high^. In contrast, cell lines with an invasive signature displayed increased motility and migration, expressed genes involved in microenvironment modulation. The recent finding that MITF^low^ cells are intrinsically resistant to targeted therapy suggests that acquisition of an invasive phenotype results in resistance to MAPK inhibitors (112).

Proliferative melanoma cells could even adopt invasive characteristics upon MAPK inhibition (115), indicating that targeted therapy could promote phenotype switching, potentially resulting in disease progression. This hypothesis is supported by the discovery that BRAF inhibition can induce invasion and metastasis *in vivo* when tumors become resistant to therapy (115). Therefore, melanoma cells may use phenotype switching as a mechanism to evade growth arrest. On this regard, the reduced sensitivity to BRAF and MEK inhibition is a clear indication that invasive cells are less dependent on cell-regulation by MAPK activity. Accumulation of several genetic and/or epigenetic alterations may participate in reverting the phenotype and allowing survival in the presence of BRAF or MEK inhibition. Current ­treatment strategies are aimed at eradicating the most proliferative cells, which may not necessarily be the most malignant. Future strategies to identify novel drugs and active treatments will have to take into consideration that both proliferative and invasive populations should be targeted. Anyway, phenotype switching is a phenomenon that cannot be ignored, especially if some treatments might induce switching to the invasive phenotype, thereby promoting metastasis formation.

## Alternative Pathways in Melanoma Growth

As previously stated, cancer genomic analysis has revealed a marked heterogeneity within tumors (116). Although some common alterations (mutations in oncogenes and tumor suppressor genes) exist, the pattern of alterations in many other genes is highly variable between individual tumors, resulting in considerable intra-tumor heterogeneity (117). This heterogeneity may represent the result of events that are positively selected during tumor progression and are influenced by a variety of factors, including the host’s genome, epigenetic changes, tumor microenvironment, immunologic features, and therapeutic interventions. Metastatic lesions, although originating from one or more subclones in primary tumor, continue to evolve after colonization of distant sites, thereby developing even greater levels of intra-tumor heterogeneity. Such heterogeneity raises a number of important issues. Clinically, intra-tumor heterogeneity has implications for detection of genetic events and for targeted therapy. Tumor sampling may have a major influence on whether all genetic alterations within a tumor will be detected, and therefore, on whether the most appropriate therapy will be offered to patients. Furthermore, genetic alterations that confer resistance to certain targeted therapies may only be present in specific subclones within a tumor mass, and if they are not detected, the patient may be subjected to inappropriate therapies.

## Autophagy

The wholeness of the signaling network that protects the integrity of the genome during the cell cycle is a fundamental blockade against cell transformation. Additional protective mechanisms are senescence, apoptosis, and autophagy. Therefore, an extensive analysis of these events in cancer cells offers the opportunity to develop new strategies for cancer control. Autophagy is a fundamental process in normal and tumor cells in response to a variety of external stimuli (118). However, the role of autophagy is still controversial; it may represent a mechanism of protection or promotion of cell death. In addition, the significance of autophagy in cancer cells is also underlined by the consideration that the response of some types of cancer cells to chemotherapy and radiotherapy is mediated by the activation of it, indicating a potential benefit an induction of autophagic cell death in cancer treatments.

Autophagy is a main lysosome-dependent process for the elimination of damaged or dysfunctional cellular components. In most cells, this self-digesting mechanism is active at a basal rate to maintain the balance between synthesis, degradation, and recycling of its constituents (119). Induction of autophagy leads to formation of an isolated membrane, the phagophore, from which it derives a double membrane cytoplasmic structure called an auto phagosome (120). The vesicular structure contains cytoplasmic material, which fuses itself with the lysosome, leading to the formation of an autophago-lysosome. Finally, the enclosed cytoplasmic content is degraded and catabolites are recycled (120). Autophagy is highly regulated; it is retained at a basal level and induced when necessary (121). In melanocytic cell lineage, autophagy regulators control many cellular trafficking pathways including melanin synthesis and melanosome formation. Autophagy has also a role in removing melanosomes in melanoma (121).

Multiprotein complexes are responsible for vesicle initiation, elongation, and fusion with the lysosome (120, 121). The initial step of autophagy involves ULK1/2 kinases, negatively regulated by mTOR. The inactivation of ULK1 in mammalian cells is sufficient to inhibit autophagy induced by amino acid deprivation (122). On the contrary, regulators inducing autophagy include tumor suppressors, such as PTEN, TSC1, TSC2, and DAPK; stress-activated signaling molecules, such as cJun N terminal kinase (JNK); and those that respond to low energy, such as the AMPK. Inhibitors of PI3K, AKT, RAS, mTORC1, and Bcl2 are able to inactivate autophagy (122). At present, it has been established that autophagy is involved in many biological functions, such as cell survival, apoptosis, metabolism, development, aging, and immunity, contributing to the etiology of many complex diseases, such as cancer, neurodegenerative, and metabolic disorders (121).

### Metformin and autophagy in melanoma

The most widely used oral antidiabetic drug metformin belongs to the family of biguanide drugs and it has been shown to inhibit the energy-sensitive AMPK target, leading to reduced protein synthesis and cell proliferation (123). Metformin was reported to act as an efficient anticancer drug in various tumors (124) as well as to inhibit the proliferation and invasion of melanoma cells (125).

Metformin inhibits mitochondrial oxidative phosphorylation, causing a decrease in ATP synthesis and an increase in the intracellular levels of adenosine monophosphate (AMP) (126, 127). The reduction in cellular energy charge causes metabolic stress that induces the activation of AMPK. This signaling pathway down-regulates processes that consume energy and activates processes that generate ATP, in keeping with the physiological role of AMPK as a primary regulator of cellular energy homeostasis (128). Activated AMPK phosphorylates downstream proteins, resulting in stimulation of catabolic pathways that generate ATP (such as glucose uptake, glycolysis, fatty acid b-oxidation, and mitochondrial biogenesis) and suppression of anabolic pathways, depending on supply of cellular ATP (such as gluconeogenesis, glycogen, cholesterol biosynthesis, protein, and fatty acid synthesis) (123, 128). In addition to such effects on lipid and glucose metabolism, AMPK – through interference with the AKT–mTOR pathway (Figure 1) – is implicated in other cellular processes, such as cell growth and proliferation, cell-cycle regulation, apoptosis, and autophagy (129). Overall, metformin, as an activator of AMPK and an inhibitor of mTOR, may stimulate autophagy. In colon cancer cells and in xenograft models, metformin induces autophagy via AMPK (130). One study also highlighted the role of p53 in the induction of autophagy (131).

Therapeutic approaches using different agents able to reprogram energy metabolism are particularly attractive. AMPK activators like metformin or phentformin have been reported to inhibit the proliferation of transformed cells (123, 132–134). In particular, metformin induces cell-cycle arrest in the G0/G1 cell-cycle phase and a strong inhibition of cell viability by induction of autophagy and apoptosis in different melanoma cells independently of the BRAF or NRAS mutational status (132). In *BRAF*-mutated melanomas, metformin used in combination with the BRAF inhibitor vemurafenib has shown a synergic antitumor effect to induce melanoma cell death (135). Furthermore, it has been reported that when another biguanide, phenformin, may cooperate with vemurafenib or classical chemotherapies in inhibiting the growth of melanoma cells (134, 136). This combined therapy could bypass the resistance found in response to BRAF^V600E^ inhibitors. However, precautions must be taken with the use of phentformin in humans. Indeed, this biguanide is reported to have more toxic effects compared with metformin due to an unacceptable incidence of severe lactic acidosis in treated patients (124).

Treatment of normal human melanocytes that express endogenous AMPK with metformin did not affect cell viability, suggesting that metformin activity is restricted to transformed cell lines and likely reflects a tumor specific regulation (123, 133). In melanoma cells, metformin can inhibit mTOR independently of AMPK activation and induces cell-cycle arrest, autophagy, and cell death. Moreover, metformin activates AMPK/p53 axis, to promote inhibition of melanoma metastasis, as well as induces down-regulation of NF-kB/STAT3 pathway, to inhibit melanoma-initiating cells (MIC) (130, 131, 137). On this regard, MIC seem to be constituted by subclones in transition to proliferative phenotype (see above), which may be at the origin of melanoma metastasis (138). This could represent a further indication that cells within the population of tumor cells in growing melanomas do not display the same tumorigenic potential.

Metformin inhibits melanoma invasion and metastasis formation in mice, through dose-dependent inhibition of the expression of proteins involved in epithelial–mesenchymal transition (EMT) (139). In particular, metformin inhibits transcription factors required for controlling the expression of genes involved in EMT, such as N-cadherin, SPARC, or fibronectin (139). Again, this process is dependent on activation of AMPK and tumor suppressor protein p53 (130, 131, 139). Furthermore, it is well established that melanoma cell invasion depends on expression of matrix metalloproteinases 2 and 9 (MMP-2 and MMP-9, respectively) (140). Both MMPs are highly expressed in melanoma cells, and a direct relationship between melanoma progression and MMP expression has been established (140). Moreover, inhibition of MMP activity has been previously investigated as a new therapeutic strategy to control metastatic spreading. Metformin reduces global MMP activity and, more specifically, expression and activity of MMP-2 and MMP-9. Decreasing EMT protein expression and MMP inhibition may thus represent the main mechanisms by which metformin negatively regulate melanoma invasiveness.

Taking into account, the drastic effect of the metformin on melanoma cell growth, survival, invasion, and *in vivo* metastasis development, it might be worth evaluating the treatment with this biguanide in patients with metastatic melanoma.

## Additional Mutated Genes

Using new sequence-based approaches to comprehensively scan the genome for non-coding mutations with potential functional impact, mutations in the telomerase reverse transcriptase (*TERT*) promoter, which encodes the catalytic subunit of telomerase, were identified at high frequencies in cutaneous melanoma (141). *TERT* promoter mutations were found to have a UV signature and to lead to an increased *TERT* gene expression, being associated with poor prognosis in melanoma patients (141). Two recent reports described activating mutations in *TERT* promoter in up to 71% of cutaneous melanomas (142, 143). The mutations result in a two to fourfold increase in gene expression. Telomerase overexpression allows neoplastic cells to continuously proliferate without entering senescence or apoptosis by maintaining telomere length and avoiding chromosomal instability. Further studies are required to fully elucidate the role of these mutations in melanoma.

Germline mutations in *BAP1* (located at 3p21) were reported to predispose to melanocytic tumors, including uveal and cutaneous melanomas (144). In addition, co-segregation with a germline mutation in *TERT* promoter has been observed in an informative melanoma-prone family (142), suggesting that this gene may also act as a rare high-penetrance melanoma susceptibility gene. Together, these genes account for melanoma susceptibility in a small proportion of melanoma-prone families.

A rare mutation (p.Ser270Asn) with founder effect in the *protection of telomeres 1* (*POT1*) gene has been identified in melanoma-prone families (145). Carriers of this variant had increased telomere lengths and numbers of fragile telomeres, suggesting that such a variant perturbs telomere maintenance. *POT1* is a component of the telomeric shelterin complex that directly binds with high specificity to single-stranded telomeric repeats (146). *POT1* prevents inappropriate processing of exposed chromosome ends by DNA damage response pathways and regulates telomerase function, thereby having a critical role in maintaining telomere integrity and regulating telomere length (147). *POT1* is a susceptibility gene for familial melanoma in other populations; *POT1* variants were indeed found in an independent study of melanoma-prone families from the UK and Australia (148). Although the real role of POT1 in melanoma – as for TERT – needs to be fully understood, all these findings suggest that genes involved in telomere maintenance may contribute to the disease pathogenesis.

## Epigenetic Targets

New sequencing approaches have also unveiled a number of mutations in genes coding for chromatin-remodeling proteins. Chromatin remodeling often involves modification of histones, by addition or removal of covalently bound methyl, acetyl, or ubiquitin residues as well as by ATP-dependent remodeling of nucleosomes (149). One chromatin-remodeling group that is involved in melanoma development and progression is the SWI/SNF complex (150). Inactivating mutations in SWI/SNF family member genes (*ARID1A*, *ARID1B*, *ARID2*, and *SMARCA4*) and in members of another chromatin-remodeling family referred to as the poly comb complex (*EZH2*, *BMI1*, and *JARID1B*/*KDM5B*) have been found altered in melanoma (107, 151–153). The role of chromatin-remodeling proteins in cancer is not fully understood. One of the most widely accepted theories is that alterations of these proteins result in cellular de-differentiation (149). Potentially, reversion of such alterations using therapeutic agents, thereby forcing cancer cells to regain a differentiated state, may prove to be clinically valuable. The role of epigenetic factors in melanoma pathogenesis is yet to be fully elucidated. In addition, alterations in DNA methylation – as hypo-methylation, leading to aberrant gene expression, and focal CpG island hyper methylation, which is generally associated with gene down-regulation – have been described (154). Non-coding RNAs, both short (e.g., microRNAs) and long, are known to be aberrantly expressed in melanoma and play as yet incompletely defined roles in pathogenesis (155). Novel compounds targeting these alterations are in development.

Epigenetic manipulation is a novel approach to cancer therapy that remains to be further explored in solid tumors (156). Epigenetic alterations may contribute to melanomagenesis by down-regulating tumor suppressor genes, apoptotic factors, and DNA repair enzymes as well as by participating in resistance mechanisms to therapies (157). Epigenetic drugs also seem to favor the endogenous antitumor immune response via several mechanisms (158). Furthermore, epigenetic drugs have shown the ability of restoring apoptotic processes that, when deregulated, appear crucial in the resistance to chemotherapeutics, immune responses, and targeted agents, such as BRAF and MEK inhibitors (88, 156, 158). Epigenetic mechanisms represent an important challenge for melanoma treatment.

Histone modifications are increasingly being involved in chromatin structure and gene expression regulation. In particular, EZH2, the catalytic subunit of the polycomb repressive complex 2 (PRC2), is overexpressed in many different types of cancers including melanoma, where it represses tumor suppressor genes (153). EZH2 is an important driver of melanoma progression and EZH2 inhibitors promising therapeutic agents. Several epigenetic drugs including the clinically used inhibitors of DNA methyltransferases (DNMTi) and histone deacetylases (HDACi) as well as the most recently discovered inhibitors of EZH2 are available and may be used in combination with immune and targeted therapies (159).

## Aurora Kinases

The aurora kinase family in mammals includes aurora kinase A (AurkA), B (AurkB), and C (AurkC) (160). They have many roles in the regulation of cell division, ensuring proper chromosome assembly, formation of the mitotic spindle, and cytokinesis. AurkA is particularly important for regulation of microtubule nucleation at spindle poles, whereas AurkB is essential for chromosome condensation, kinetochore function, cytokinesis, and the proper function of the spindle assembly (161). AurkA has long been recognized as an oncogene, due to its overexpression and amplification in several human cancers; however, it is unlikely to act as oncoprotein, since its over expression neither transforms primary cells nor leads to tumor formation (162). Overexpression of the aurora kinases induces aneuploidy and genomic instability, which have a leading role in the pathogenesis of various tumors (160). Nonetheless, their powerful roles in cell-cycle regulation and suitability for inhibition by small-molecule antagonists make both AurkA and AurkB promising anticancer therapy targets.

AurkA associates with many other key targets involved in tumorigenesis (163). Functionally interacting proteins include MYC, NF-kB, AKT1, and p53 (see Figure 1). Phosphorylating activity by AurkA removes inhibition of nuclear factor NF-kB, supporting transcription of pro-survival genes, and stimulates cell migration (164). Overexpression of AurkB, whose main partner is the survivin protein, is associated with poor outcomes in colon cancer, anaplastic thyroid cancer, and glioblastoma (165). Finally, both AurkA and AurkB associate with polo-like kinases and other additional partners crucial for oncogenic activity (162). For example, up-regulation of both AurkA and AurkB can contribute to cancerogenesis through the phosphorylation of p53, which accelerates MDM2-dependent degradation of the same p53 protein by the poly-ubiquitination-proteasome pathway (166). By contrast, AurkA activity is suppressed by p53 binding, leading to an increased AurkA activation in p53-mutant tumors (89).

Overexpression of these kinases has been observed in several tumor types – including carcinomas of the colon, breast, prostate, pancreas, thyroid, and head and neck – being associated with advanced clinical stage and poor prognosis (89). Inhibition of AurkA protein, which seems to be expressed at high levels in melanoma (163), has been shown to limit tumor growth, impair mitosis, and induce senescence in melanoma, suggesting a potential role as therapy target (167). AurkA inhibitor enhanced the effect of B-RAF and MEK inhibitors on melanoma cell growth in a 3D human skin reconstruction model (167). Combined BRAF/AurkA inhibition might offer a therapeutic alternative to BRAF/MEK inhibition for *BRAF*-mutated melanomas, while a combination of MEK/AurkA inhibitors could represent a possible option for patients without *BRAF* mutations. Moreover, a triple drug combination including inhibitors of BRAF, MEK, and AurkA offered increased efficacy against melanoma cell growth (167); it might become a potential innovative treatment strategy.

## Future Perspectives

The introduction of novel techniques for genetic analyses may lead to a much better understanding of the mechanisms involved in the pathogenesis of melanoma. Assays for assessing the variations in DNA copy number have been introduced in clinical practice and are now commonly used to assist pathologic distinction between benign and malignant melanocytic lesions. A panel of molecular tests based on mutation analyses in targetable effectors of the main pathways underlying development and progression of melanoma (particularly, components of the MAPK signaling cascade) – using next-generation sequencing (NGS) approaches – is being utilized for predicting purposes (168). Individual targeted therapies have shown promising early responses, but these encouraging results are mitigated by the rapid and frequent emergence of resistance (169). Combining immunotherapies and small inhibitor therapies could potentially alleviate the shortcomings of individual agents, leading to more durable responses, and, subsequently, longer survivals in higher numbers of patients (170). To date, in patients with melanomas harboring genetic alterations for which specific targeted therapies are not available, immunotherapies may be promising therapeutic options (171). Overall, considerable research is under way to elucidate which molecular mechanisms possess a clinical relevance; this could be helpful to also explore mechanisms of resistance and develop strategies to prevent or circumvent them.

Detailed genetic analysis in terms of massively parallel sequencing is rapidly evolving from a research tool to a potentially useful and widespread clinical standard for the management of cancer patients, particularly as the number of available targeted agents’ increases. The aim is to quickly translate successful experimental therapeutic approaches to the clinic, with patients receiving a personalized selection of the tested therapeutic modalities based on the sequencing profiles of their tumors. Large sequencing studies have identified several novel genetic alterations in melanoma (172), and additional mutations will inevitably be identified.

Important challenges will be to personalize and combine available therapeutic options based on anticipated mechanisms of resistance relevant to each tumor. Results from NGS-based analysis of multiple melanoma biopsies obtained before treatment, during response to therapy, and after disease progression are starting to emerge, revealing important aspects of *in vivo* resistance mechanisms. The discovery of new therapeutic compounds is also awaited for a further improvement in treating melanomas that are resistant to existing therapies. Efforts to improve durability of responses will likely include double, triple, and even quadruple drug regimens.

Nevertheless, strategies to combine the most effective targeted treatments with cancer immunotherapy, which is becoming the standard of care in advanced melanoma, will be useful to both improve the clinical management and achieve much longer survivals among patients with such a disease.

After a very long period of dormancy in melanoma treatment, a new era of successful molecular-based therapeutic strategies has begun.

## Author Contributions

GP, conceived of the study, drafted the manuscript. MO, drafted the manuscript. MCo, MCa, MS, and AM, performed data collection and interpretation. PP, prepared all Figures. PA, revised manuscript critically. AC, performed final approval of the manuscript.

## Conflict of Interest Statement

Paolo Antonio Ascierto is consultant of Bristol Myers Squibb, MSD, and Roche-Genentech. He participated in the Advisory Board for Bristol Myers Squibb, MSD, Roche-Genentech, GSK, Amgen, Celgene, Medimmune, and Novartis. He received honoraria from Brystol Myers Squibb, MSD, and Roche-Genentech. All remaining authors declare the absence of any Conflict of Interest.
